# Our Experience with Transcatheter Aortic Valve Replacement Medication Outcomes from 2013 to 2016

**DOI:** 10.7759/cureus.6030

**Published:** 2019-10-30

**Authors:** Robert Herron, Aravinda Nanjundappa, Frank H Annie, Megan Wood, Sarah Embrey, Carly Heatherly, Alfred Tager

**Affiliations:** 1 Surgery, Charleston Area Medical Center, Charleston, USA; 2 Cardiology, Charleston Area Medical Center, Charleston, USA; 3 Heart and Vascular Center, Charleston Area Medical Center, Charleston, USA; 4 Pharmacy, University of Charleston School of Pharmacy, Charleston, USA; 5 Osteopathic Medicine, West Virginia School of Osteopathic Medicine, Buckhannon, USA; 6 Emergency Medicine, Charleston Area Medical Center, Charleston, USA

**Keywords:** structural heart disease, tavr

## Abstract

Background

Aortic stenosis is classified as stenosis that can be caused by a congenital disability in infants and children but is more commonly produced by a degenerative process of calcification and scarring of the valve in the later decades of life. High systemic pressure and hemodynamic disturbances characteristic of this area of the cardiovascular system makes the aortic valve susceptible to plaque and cholesterol buildup over time, similarly to atherosclerosis, contributing to the pathology of aortic stenosis. Thus, this study aims to assess the short and long-term clinical outcomes of risk factor reduction, post transcatheter aortic valve replacement (post-TAVR), and results of tested medication outcomes.

Methods

Data were obtained from Charleston Area Medical Center, which is a tertiary care 800-bed community teaching facility and was examined using STATA 11.4 (StataCorp LLC, College Station, Texas, USA), a Cox proportional hazards model to test for clinical significance. This study examined the medications aspirin, clopidogrel, beta-blockers, and angiotensin-converting enzyme (ACE) inhibitors. Additional medications analyzed included statin, anticoagulant, aspirin with clopidogrel, and beta-blocker with ACE inhibitor and statin following the procedure of transcatheter aortic valve replacement (TAVR) and the overall risk of a hazard event of mortality.

Results

Results suggest that clopidogrel by itself had the lower rate of mortality at one year with hazards of 0.6906, a p-value of 0.221 and a 95% confidence interval of 0.3677 - 1.259; and at three years with hazards of 0.4845, a p-value of 0.027 and a 95% confidence interval of 0.2552 - 0.9201. Statins had the second-lowest rate at one year with hazards of 0.7299 and a p-value 0.215 and a 95% confidence interval of 0.4438 - 1.200; and at three years with hazard of 0.8529 and a p-value of 0.530 and a 95% confidence interval of 0.5192 - 1.401. Both of these medications had a consistent lower hazard and/or risk of death compared to other standard medication regiments. Within our center's data, clopidogrel had the best clinical outcome.

Conclusions

This study showed that therapy with aspirin and clopidogrel alone did not demonstrate a significant increase in mortality versus alternative anticoagulation therapy in patients post aortic valve replacement. Clopidogrel and statin usage post-aortic valve revascularization may have a trend towards a reduction in mortality.

## Introduction

As the aortic valve begins to calcify, less blood can be pumped efficiently from the left ventricle of the heart to the aorta. This lack of blood directly affects the blood supply to the brain as well as the rest of the body causing symptoms characteristic of aortic stenosis. These symptoms vary among patients but typically include angina, shortness of breath, syncope or lightheadedness, and congestive heart failure. Also, as a result of the aortic valve being narrowed, the pressure gradient between the left ventricle and the aorta increases. The left ventricle compensates for the restricted blood flow, and this overworking mechanism can cause hypertrophy of the myocardium. Symptomatic aortic stenosis narrowing of the valve along with a difference in the mean gradient of 40 mm of Hg between the left ventricle and aorta is necessary for the diagnosis and treatment of aortic stenosis. The severity of aortic valve stenosis is classified into three categories: mild, moderate, and severe [1]. These categories are based on three variables: the valve area, the peak aortic valve velocity, and the mean pressure gradient.

Treatment options vary for patients with aortic stenosis. Some asymptomatic patients may be considered safe to monitor only, but typically the valve should be replaced. Currently, the indications for aortic valve replacement include severe, symptomatic aortic stenosis. This represents a Class I evidence recommendation for aortic valve replacement. For the patient with aortic stenosis that is asymptomatic, the Class I data for replacement include severe aortic stenosis with a left ventricular ejection fraction less than 50% and for severe aortic stenosis in patients who are undergoing cardiac surgery for a separate pathology (i.e., coronary artery bypass grafting). The Class IIa evidence that supports aortic valve replacement in the asymptomatic patient includes patients with very severe stenosis (aortic velocity > 5m/s) who are considered low surgical risk [2]. Also, aortic valve replacement may be indicated in asymptomatic patients with severe aortic stenosis who experience a decrease in exercise tolerance or a decrease in blood pressure during exercise. Another indication for valve replacement in the asymptomatic patient is severe stenosis with rapid disease progression, which is defined by an increase in aortic velocity equal to or higher than 0.3 m/s per year. This particular indication for aortic valve replacement is a Class IIb indication [3].

As transcatheter aortic valve replacement (TAVR) therapy becomes increasingly prevalent, postoperative pharmacologic treatment is also a very high yield area of research for determining the optimal care plan for these patients. Heart condition patients have typically prescribed a variety of medications to promote adequate blood perfusion and limited coagulation. These medications include aspirin and clopidogrel for antiplatelet activity as well as beta-blockers and angiotensin-converting enzyme (ACE) inhibitors for the effects on regulating blood pressure [4]. Anticoagulants may also be used to decrease blood clot formation in patients with artificial valves, depending on the type of valve utilized. Statins, including atorvastatin, rosuvastatin, and pravastatin help reduce cholesterol levels. Combination therapy of aspirin and clopidogrel has shown to be statistically relevant for lowering mortality post aortic valve replacement. Statin therapy has also shown to be associated with an improved survival time when prescribed for aortic valve replacement patients [5]. This research study intended to compare different combinations of typically prescribed medications and the risk of the average hazards associated with mortality. 

## Materials and methods

Patients that received an aortic valve replacement in the form of a transcatheter aortic valve replacement between the period of January 1, 2013, and December 31, 2016, were included. The total sample size of TAVR was (n=150). Data collected from the data warehouse included account number, a medical record number, admission and discharge dates, gender, age, procedure code, and last point of contact. Further data collected from each patient chart, including past medical history, included cardiac resynchronization therapy, diabetes, coronary artery disease, and congestive heart failure status. Medications prescribed at discharge, including aspirin, clopidogrel, beta-blocker usage, ACE inhibitor usage, statin usage, and anticoagulant usage, were also collected for analysis. This data was then compiled into an excel spreadsheet with “yes” indicated by a one and “no” indicated by a zero to be used for statistical analysis. STATA/SE 11.4 (StataCorp LLC, College Station, Texas, USA) program was used to analyze the data. A Cox proportional hazards model was applied to calculate the hazard ratios regarding mortality by using the last point of contact as an indicator of death. Results of the hazard ratio for the combination of aspirin and clopidogrel were compared to new anticoagulant therapy over 365 days as well as the entire period of the study.

Results of the hazard ratio for the combination of aspirin and clopidogrel were also compared to the combination of a beta-blocker, ACE inhibitor, and a statin over 365 days as well as for the length of the study. Into graphs to visually represent the hazard comparisons over time, a p-value was calculated to compare to the standard 0.05 to determine the significance of the results. The Cox proportional hazards model investigated the effect of multiple risk factors on the time mortality would take to occur. The predictors have an equivalent impact on the expected risk. 

## Results

**Figure 1:**
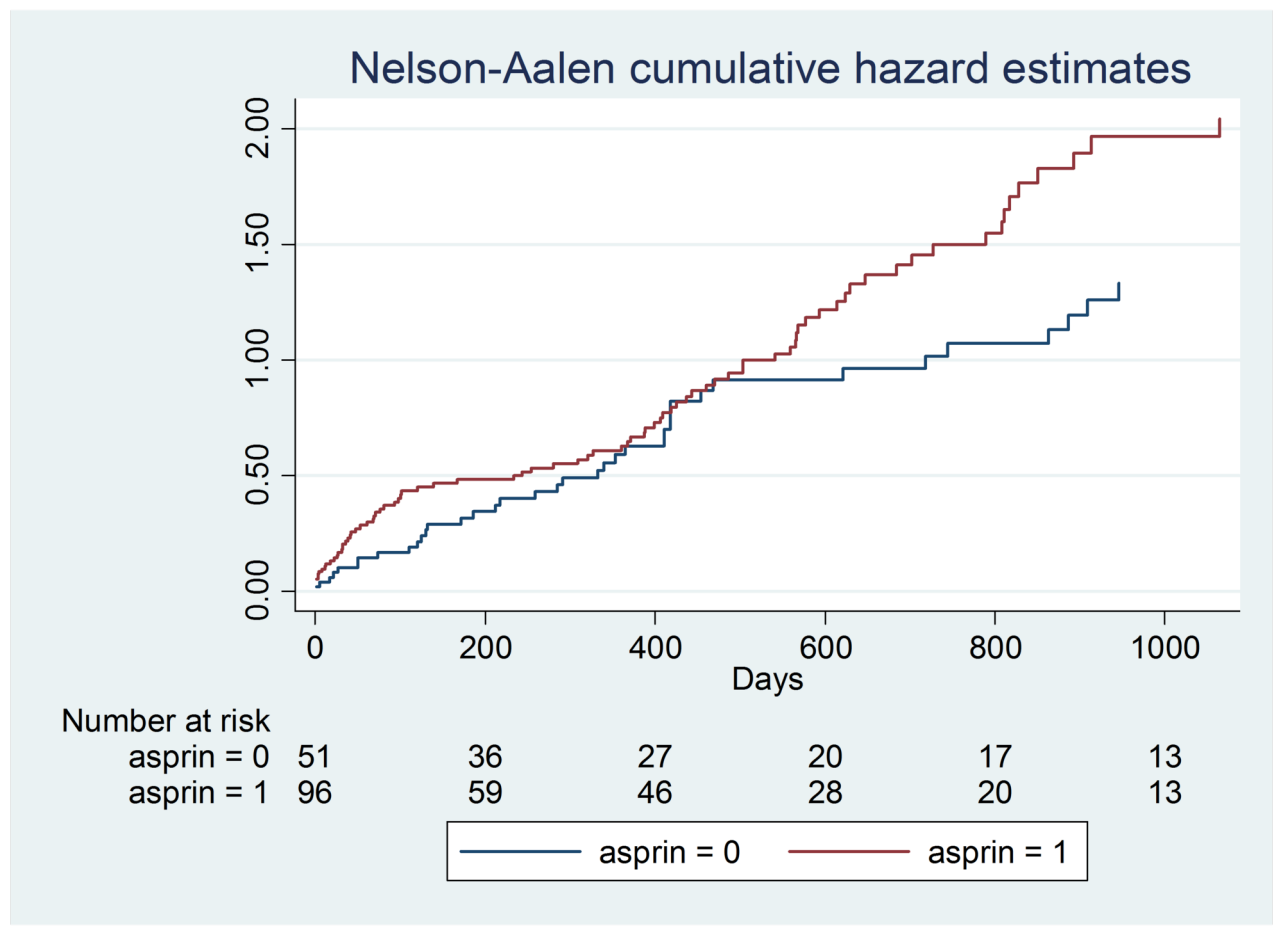
Aspirin hazard ratios

**Figure 2:**
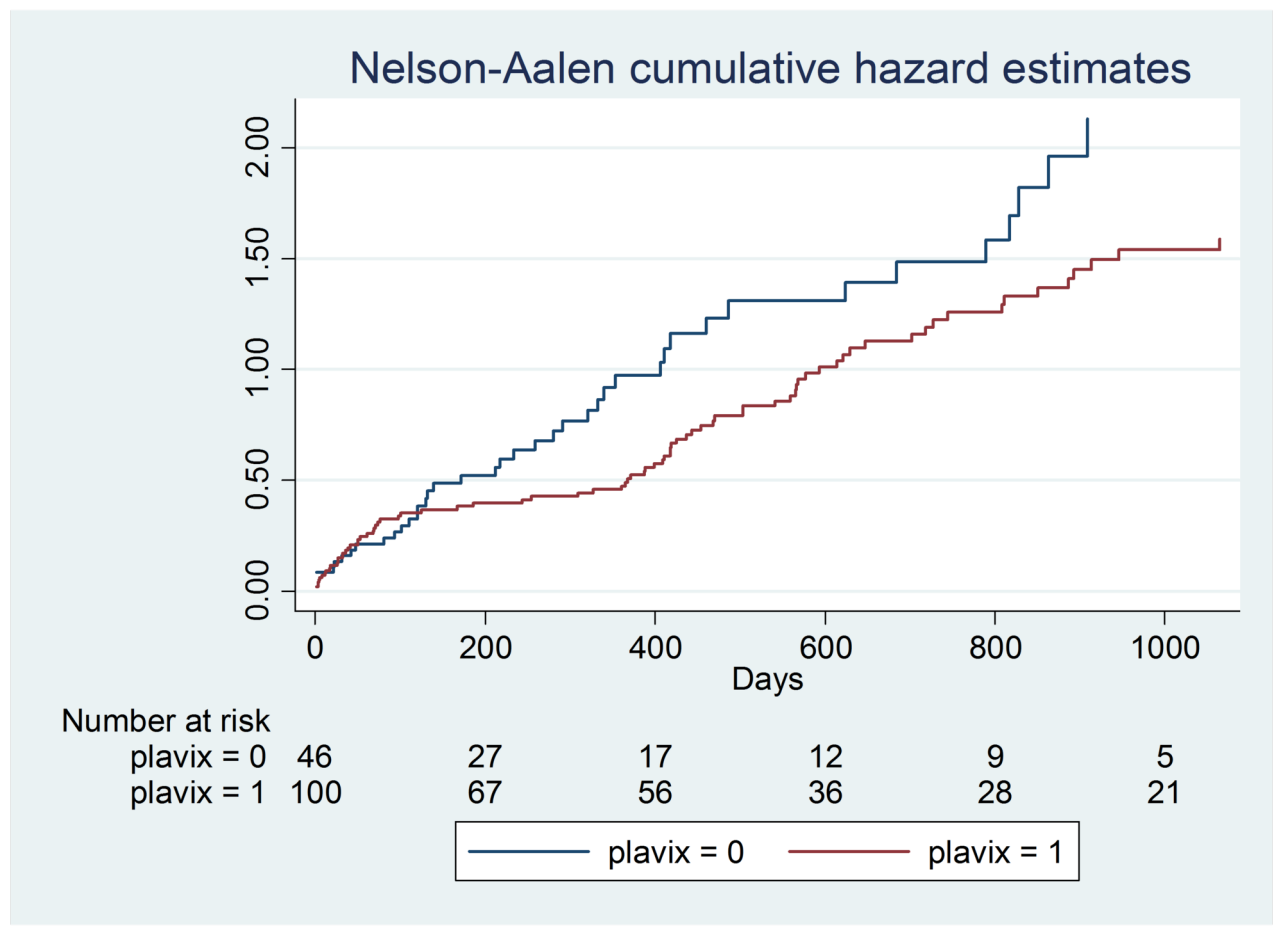
Clopidogrel hazard ratios

**Figure 3:**
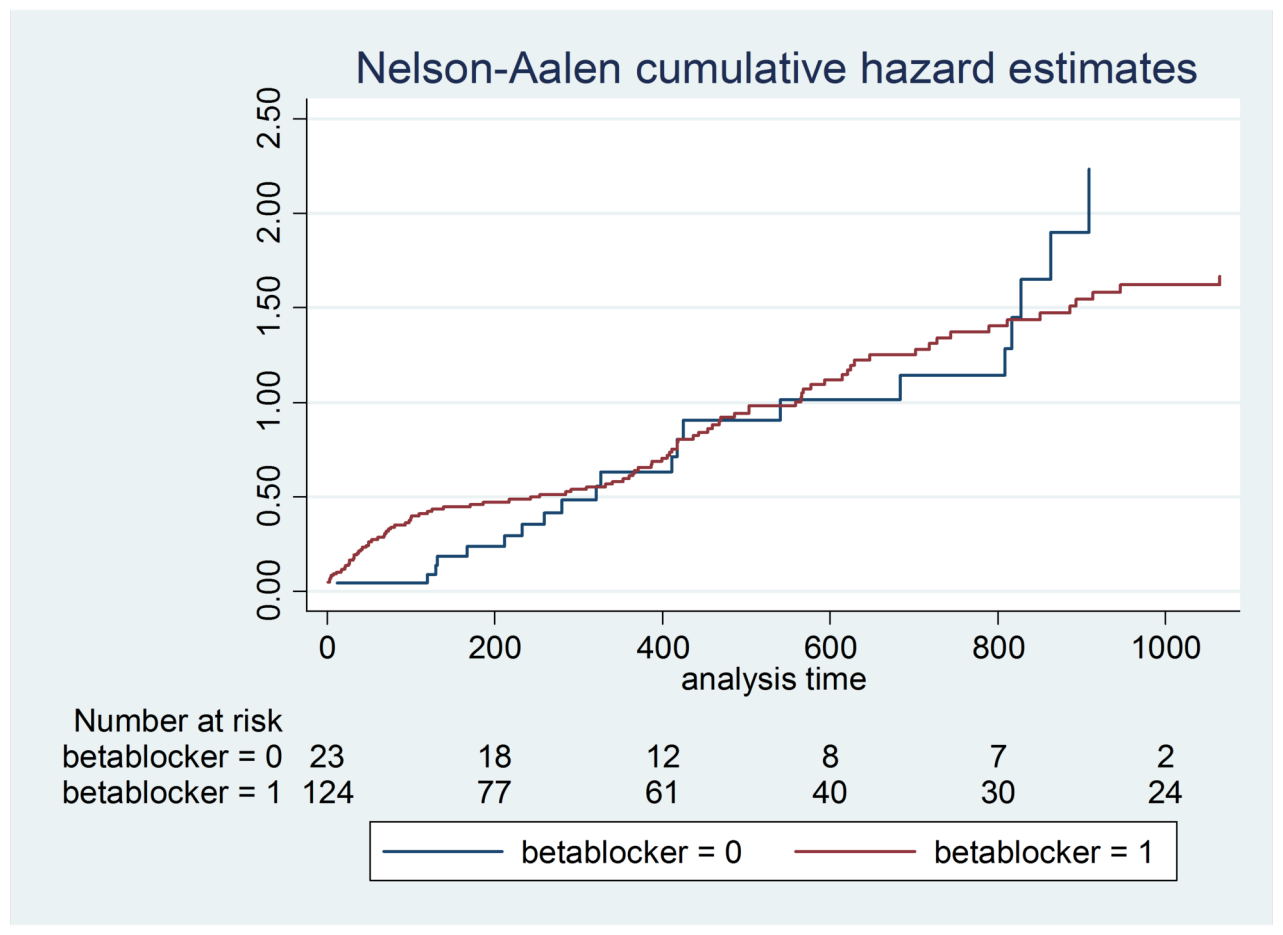
Beta-blocker hazard ratios

**Figure 4:**
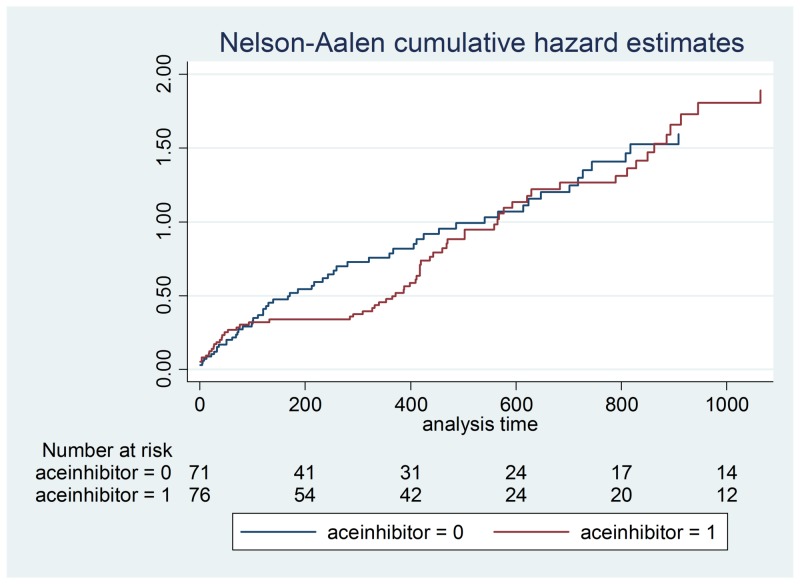
ACE inhibitor hazard ratios ACE - angiotensin-converting enzyme

**Figure 5:**
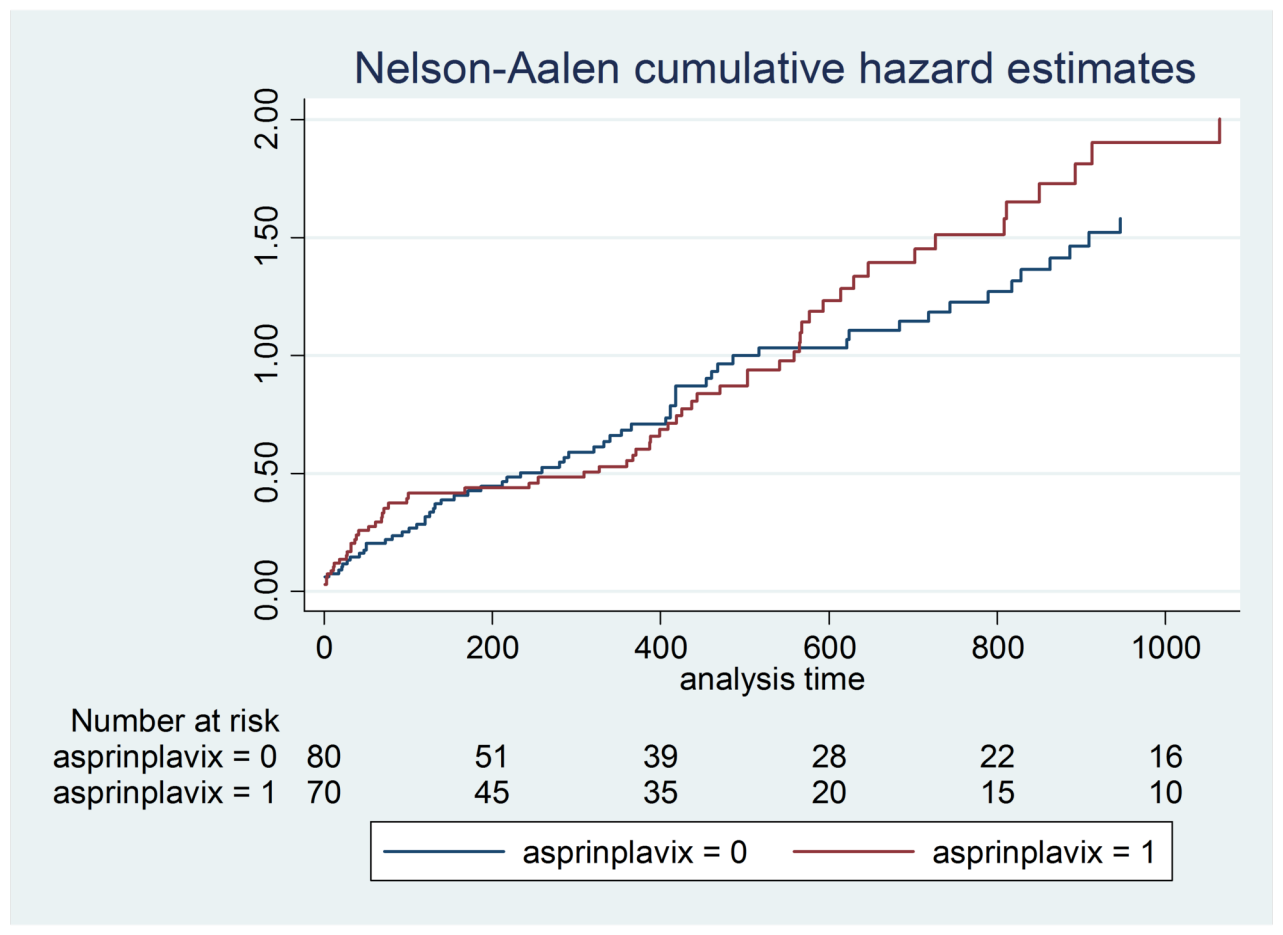
Aspirin + clopidogrel hazard ratios

**Figure 6:**
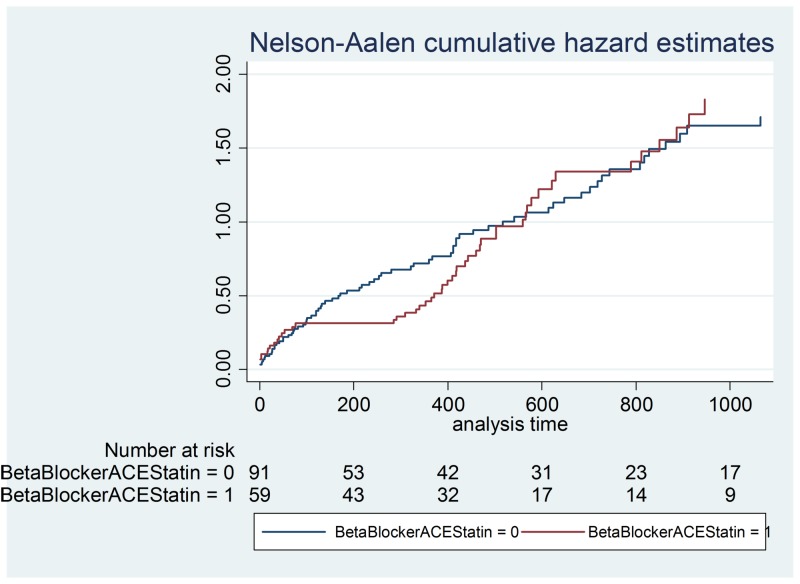
Beta-blocker + ACE inhibitor + statin hazard ratios ACE - angiotensin-converting enzyme

**Figure 7:**
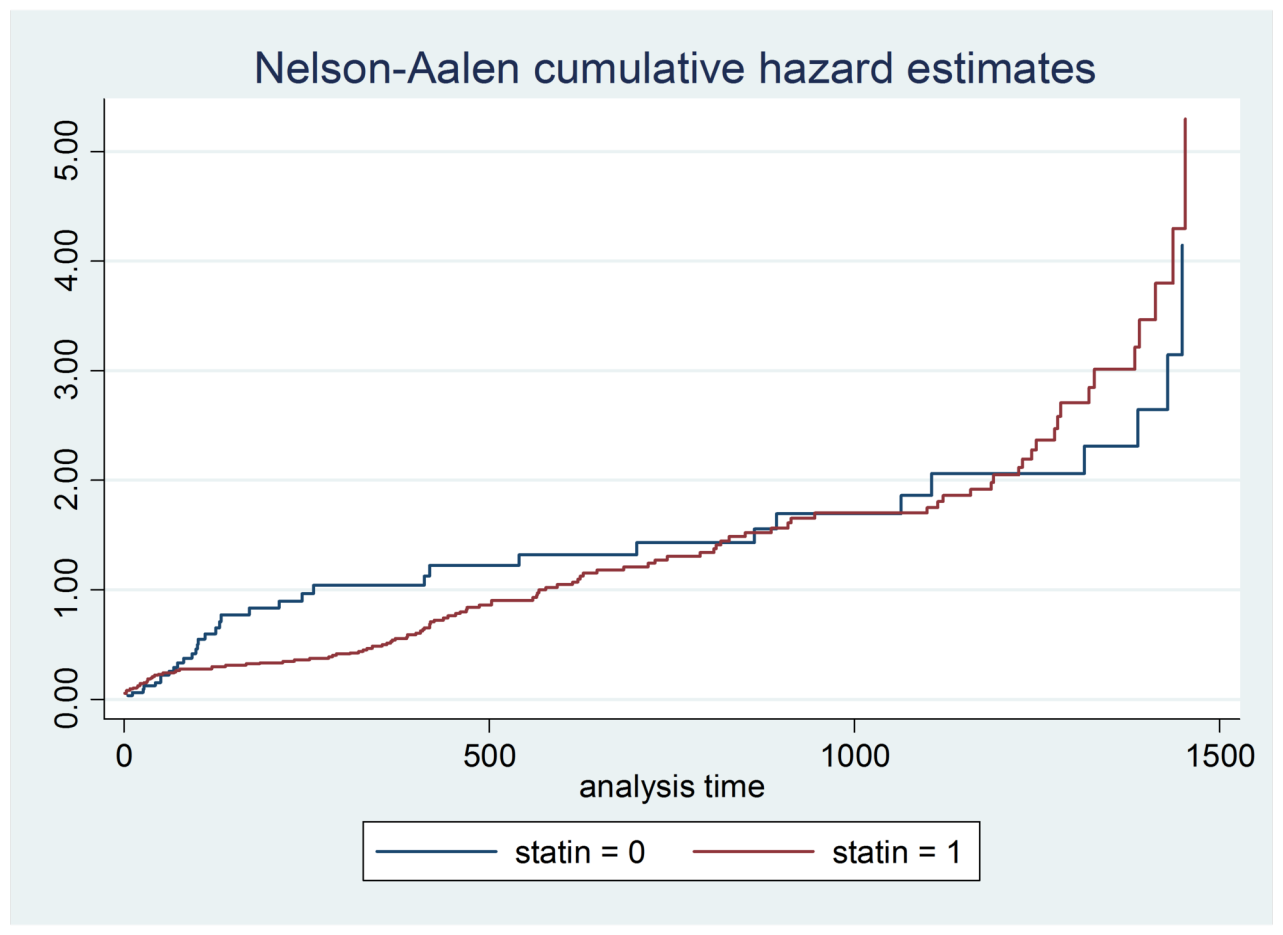
Statin hazard ratios

Demographic data is illustrated in Table 2 and a Cox proportional hazards model outputs are shown in Table 3-4. Figures 1-7 demonstrate that clopidogrel and statin usage have the lowest hazard at one and three years. At one year, clopidogrel had hazard ratio of 0.6806 (p=0.221) and a 95% confidence interval of 0.3677 - 1.259, which slowly increased over the first year of the study. The medication of statin usage had a hazard ratio of 0.7299 (p= 0.215) and a 95% confidence interval of 0.4438 - 1.200 which only had a slightly higher hazard ratio than clopidogrel at this stage of the study. At three years of research, clopidogrel had hazards of 0.4845 (p=0.027) and a 95% confidence interval of 0.2552 - 0.9201, which was statistically significant. Statin usage had a hazard ratio of 0.8529 (p=0.530) and a 95% confidence interval of 0.5192 - 1.401. Overall, clopidogrel and statin usage have a reportable finding in a clinical outcome. Only clopidogrel at three years in TAVR patients had a reportable statistically significant result.

**Table 1:** Demographic data TAVR - transcatheter aortic valve replacement; ACE - angiotensin-converting enzyme; CHF - chronic heart failure; CRT - cardiac resynchronization therapy

Category	TAVR
Male	86
Female	64
18 – 30	1
31- 50	2
51 – 70	32
71 – 95	116
Aspirin	96
Clopidogrel	100
Beta-blocker	124
ACE inhibitor	76
Statin	112
Anticoagulant	41
Aspirin + clopidogrel	70
Beta-blocker + ACE inhibitor + statin	59
Diabetes	69
High cholesterol	99
Coronary disease	92
CHF	43
CRT	26

**Table 2:** Hazard ratios, p-value and 95% confidence interval (1 year results) TAVR - transcatheter aortic valve replacement; ACE - angiotensin-converting enzyme; CHF - chronic heart failure; CRT - cardiac resynchronization therapy

Category	TAVR
Male	Comparison group
Female	1.182 (p=0.362) 0.8245 - 1.696
18 – 30	NA
31- 50	Comparison group
51 – 70	0.0784 (p=0.002) 0.0155 - 0.3970
71 – 95	0.0850 (p=0.003) 0.0169 - 0.4278
Aspirin	1.142 (p=0.685) 0.6004 - 2.174
Clopidogrel	0.6806 (p=0.221) 0.3677 - 1.259
Beta-blocker	1.236 (p=0.456) 0.7079 - 2.159
ACE inhibitor	0.8132 (p=0.544) 0.4173 - 1.584
Statin	0.7299 (p=0.215) 0.4438 - 1.200
Anticoagulant	0.9224 (p=0.704) 0.6086 - 1.397
Aspirin + clopidogrel	0.9428 (p=0.884) 0.4264 - 2.084
Beta-blocker + ACE Inhibitor + statin	1.160 (p=0.703) 0.5394 - 2.497
Diabetes	1.224 (p=0.267) 0.8564 - 1.750
High cholesterol	0.8927 (p=0.565) 0.6066 - 1.313
Coronary disease	0.9039 (p=0.588) 0.6274 - 1.302
CHF	0.9916 (p=0.966) 0.6714 - 1.464
CRT	0.8057 (p=0.371) 0.5020 - 1.293

**Table 3:** Hazard ratios, p-value and 95% confidence interval (3 years results) TAVR - transcatheter aortic valve replacement; ACE - angiotensin-converting enzyme; CHF - chronic heart failure; CRT - cardiac resynchronization therapy

Category	TAVR
Male	Comparison
Female	1.0244 (p=0.897) 0.7114 - 1.475
18 – 30	0
31- 50	Comparison
51 – 70	0.0944 (p=0.004) 0.0185 - 0.4794
71 – 95	0.0821 (p=0.003) 0.0162 - 0.4158
Aspirin	1.1050 (p=0.763) 0.5769 - 2.116
Clopidogrel	0.4845 (p=0.027) 0.2552 - 0.9201
Beta-blocker	1.126 (p=0.694) 0.6223 - 2.039
ACE inhibitor	0.8765 (p=0.649) 0.4348 - 1.679
Statin	0.8529 (p=0.530) 0.5192 - 1.401
Anticoagulant	0.8765 (p=0.544) 0.5728 - 1.341
Aspirin + clopidogrel	1.262 (p=0.569) 0.5656 - 2.818
Beta Blocker + ACE inhibitor + statin	1.201 (p=0.638) 0.5598 - 2.577
Diabetes	1.207 (p=0.318) 0.8338 - 1.749
High cholesterol	0.9177 (p=0.672) 0.6169 - 1.365
Coronary disease	0.9678 (p=0.863) 0.6678 - 1.402
CHF	0.8357 (p=0.410) 0.5453 - 1.280
CRT	0.4784 (p=0.006) 0.2830 - 0.8085

 

This study showed that therapy with aspirin and clopidogrel alone did not demonstrate a significant increase in mortality versus alternative anticoagulation therapy in patients post aortic valve replacement. No significant increase in mortality was found in post aortic valve replacement patients who were treated with a beta-blocker, ACE inhibitor, and statin when compared to aspirin and clopidogrel alone. We feel that these findings could help define new treatment strategies regarding patients who have undergone aortic valve replacement. As TAVR therapy has become more prevalent, the various post-procedure medication regimens have been studied as well. The post-TAVR medication strategy currently remains controversial. The American College of Cardiology Expert Consensus for the post-TAVR adult patient undergoing the procedure for aortic stenosis recommends dual antiplatelet therapy including clopidogrel 75 mg for three to six months post-procedure, and aspirin 75-100 mg. However, some studies have compared aspirin alone versus dual antiplatelet therapy that had found aspirin therapy apart to not only be non-inferior to dual antiplatelet therapy in the post-TAVR patient but to also confer less of bleeding risk in this particular patient population [6]. 

## Discussion

The standard protocol for medication utilization in aortic valve replacement patients as indicated by the American College of Cardiology and the American Heart Association states that patients receiving a transcatheter aortic valve replacement should receive pharmacological therapy consisting of aspirin and clopidogrel for a minimum of six months post-procedure [7]. The primary indication for antiplatelet therapy in the post aortic valve replacement patient is to prevent the occurrence of a thromboembolic event, namely a stroke. The majority of periprocedural strokes arise from thromboembolism from the actual valve site. The dislodgment of atheroemboli originating from an ulcerative plaque within the great vessels during maneuvering and manipulation of the catheter can lead to a stroke as well. Due to the risk of thromboembolism complications with TAVR dual antiplatelet treatment with aspirin and clopidogrel is recommended to decrease the risk of a thromboembolic event, assuming no contraindications to these medications exist [7]. However, unlike surgical aortic valve replacement, where there is a higher quantity of experience with anticoagulation, the management of anticoagulation and antiplatelet therapy in the post-TAVR patient appears to be more variable [8-10].

This data illustrates that antiplatelet therapy with aspirin and clopidogrel alone after aortic valve replacement may, in fact, be a safe and viable alternative to anticoagulation usage in post aortic valve replacement patients [11-13]. The results not only show that dual antiplatelet treatment after aortic valve replacement is safe but also efficacious in reducing the risk of mortality in the post valve replacement patients [14]. This is very interesting to consider regarding the optimization of a medical therapy plan in this particular patient population, especially when accounting for the presumed increased risk of bleeding in patients who are also treated with anticoagulation therapy [15-20]. Our results also show that the combination of medical therapy including a beta-blocker, ACE inhibitor, and a statin did not significantly increase the mortality risk in post aortic valve replacement patients when compared to post valve replacement patients treated with aspirin and clopidogrel alone. We feel this is important as it shows that the addition of these medications to the dual antiplatelet regimen does not place these patients at an increased risk of mortality, and also confers the benefit that these particular drugs have on the patient with structural heart disease [20-22].

Our data also revealed that patients with a history of congestive heart failure who underwent aortic valve replacement had a more significant mortality risk within the first 30 days postoperatively when compared to patients who did not have these comorbidities. Thus, the fact that our study did not show any increase in mortality risk in post valve replacement patients who were prescribed a beta-blocker, ACE inhibitor, and a statin is significant because these medications are crucial in the treatment and management of these comorbidities. Our findings support that these medications are safe to administer in the post aortic valve replacement patient in addition to the aspirin and clopidogrel dual antiplatelet therapy. We feel this finding is significant because these medications are paramount in the treatment of mentioned comorbidities and thus should be continued post valve replacement.

When reviewing the results of this research study, several limitations need to be considered. First, in retrospective studies assessing mortality, the death records are not always accurate. This data was collected through a hospital data collection, and not all death records are reported to the hospital where the aortic valve replacement was conducted. For this reason, the last point of contact for each patient with Charleston Area Medical Center was used to assess mortality. This is a limitation because it does not accurately represent mortality, and may therefore not accurately describe the results of this study. Secondly, data collected for analysis regarding past medical history, social history and medications at discharge relied solely on the accuracy of the patient chart that was taken by the attending physician. If any relevant information were omitted from the chart, this would have swayed the results of the analysis. The researchers believe that the information taken in the patient charts was accurate enough to validate the obtained results.

## Conclusions

This study showed that therapy with aspirin and clopidogrel alone did not demonstrate a significant increase in mortality versus alternative anticoagulation therapy in patients post aortic valve replacement. As the TAVR procedure becomes more common differing medication protocols are necessary to tailer the differing medication combinations depending on both the patient and the patient population being treated. This evolution has been observed through the years as TAVR programs have become more significant over time, and differing medication diagnosis has been implemented and changed to fit individual cases. No significant increase in mortality was found in post aortic valve replacement patients who were treated with a beta-blocker, ACE inhibitor, and statin when compared to aspirin and clopidogrel alone within this study. We feel that these findings could help define new treatment strategies regarding patients who have undergone aortic valve replacement. 
